# Health-related quality of life in a european sample of adults with early-treated classical PKU

**DOI:** 10.1186/s13023-023-02917-w

**Published:** 2023-09-22

**Authors:** Stephanie Maissen-Abgottspon, Raphaela Muri, Michel Hochuli, Péter Reismann, András Gellért Barta, Ismail Mucahit Alptekin, Álvaro Hermida-Ameijeiras, Alessandro P. Burlina, Alberto B. Burlina, Chiara Cazzorla, Jessica Carretta, Roman Trepp, Regula Everts

**Affiliations:** 1 Department of Diabetes, Endocrinology, Nutritional Medicine and Metabolism, Inselspital, Bern University Hospital, University of Bern, Bern, Switzerland; 2 Support Center for Advanced Neuroimaging (SCAN), Institute of Diagnostic and Interventional Neuroradiology, Inselspital, Bern University Hospital and University of Bern, Bern, Switzerland; 3 Department of Internal Medicine and Oncology, Semmelweis University, Budapest, Hungary; 4 Faculty of Health Sciences, Department of Nutrition and Dietetics, Ankara University, Ankara, Turkey; 5 Division of Internal Medicine, European Reference Network for Hereditary Metabolic Disorders (MetabERN), University Clinical Hospital, Santiago de Compostela, Spain; 6 Neurological Unit, St. Bassiano Hospital, Bassano del Grappa, Italy; 7 Division of Inborn Metabolic Diseases, Department of Pediatrics, University Hospital, Padua, Italy; 8 Division of Neuropediatrics, Development and Rehabilitation, Department of Pediatrics, Inselspital, Bern University Hospital, University of Bern, Bern, Switzerland

**Keywords:** Phenylketonuria, Health-related quality of life, Inherited metabolic disease, Cognition, Metabolic control

## Abstract

**Background:**

Phenylketonuria (PKU) is a rare inborn error of metabolism affecting the catabolism of phenylalanine (Phe). To date, findings regarding health-related quality of life (HRQoL) in adults with early-treated classical PKU are discrepant. Moreover, little is known about metabolic, demographic, and cognitive factors associated with HRQoL. Hence, we aimed to investigate HRQoL and its association with demographic, metabolic, and cognitive characteristics in a large European sample of adults with early-treated classical PKU.

**Results:**

This cross-sectional study included 124 adults with early-treated classical PKU from Hungary, Italy, Spain, Switzerland, and Turkey. All participants prospectively completed the PKU quality of life questionnaire (PKU-QoL), a questionnaire specifically designed to evaluate the impact of PKU and its treatment on HRQoL in individuals with PKU. In addition, information about Phe levels (concurrent and past year), demographic (age and sex), and cognitive variables (intelligence quotient, IQ) were collected. Most domains revealed little or no impact of PKU on HRQoL and more than three-quarters of the patients rated their health status as good, very good, or excellent. Nevertheless, some areas of concern for patients were identified. Patients were worried about the guilt that they experience if they do not adhere to the dietary protein restriction and they were most concerned about high Phe levels during pregnancy. Further, tiredness was the most affected symptom, and the supplements’ taste was considered a main issue for individuals with PKU. The overall impact of PKU on HRQoL was higher in women (*U* = 1315.5, *p* = .012) and in adults with a lower IQ (*r*_*s*_ = − 0.448, *p* = .005). The overall impact of dietary protein restriction was higher in adults with higher concurrent Phe levels (*r*_*s*_ = 0.272, *p* = .007) and higher Phe levels during the past year (*r*_*s*_ = 0.280, *p* = .009).

**Conclusion:**

The impact of PKU on most domains assessed in the PKU-QoL was considered to be low. These results likely reflect the successful implementation of the newborn screening resulting in the prevention of severe adverse long-term outcomes. However, a particular clinical focus should be given to patients with lower IQ, higher Phe levels, and women, as these variables were associated with a lower HRQoL.

## Background

Phenylketonuria (PKU, OMIM 261600) is a rare inborn error of metabolism affecting the catabolism of phenylalanine (Phe) to tyrosine. Mutations in the phenylalanine hydroxylase (*PAH*) gene lead to impaired activity of the PAH enzyme, which results in elevated levels of Phe in the blood and brain [1]. An early-initiated treatment consisting of a Phe-restricted diet and amino acid supplementation successfully prevents severe long-term sequelae, including mental retardation, neurological impairment, or psychiatric difficulties [2]. Pharmacological treatments, such as sapropterin dihydrochloride (BH4, Kuvan®) or pegvaliase (Palynziq®), have been introduced, allowing patients a higher Phe intake [3]. However, a substantial amount of patients with classical PKU do not respond to sapropterin or display significant hypersensitivity reactions to the treatment with pegvaliase [3–5], leaving a Phe-restricted diet combined with an amino acid supplementation the treatment of choice for most patients with classical PKU.

Maintenance of a lifelong Phe-restricted diet is complex and adherence to treatment can be challenging for patients [6, 7]. In most adults with PKU, difficulties following dietary recommendations result in higher Phe levels than recommended according to the current guidelines [7, 8]. Issues with cognitive performance, depression, and irritability might concern adults with PKU and likely interfere with their daily lives [7]. Higher scores in depression, anxiety, or stress have been described in adults with early-treated classical PKU, and eating disorders are more prevalent than in the general population [9–11]. These PKU-related symptoms can affect health-related quality of life (HRQoL) of adults with early-treated PKU [12]. HRQoL refers to the subjective evaluation of health or illness and encompasses psychological, physical, and social domains of health [13, 14]. In addition to PKU-related symptoms affecting HRQoL, following a diet with a stringent restriction of natural protein can be stressful for patients and consequently also affects HRQoL [12]. Thus, a differentiation between PKU-related symptoms and dietary management requirements affecting HRQoL is important when investigating HRQoL in PKU [12].

Several studies suggest that health-related QoL in adults with early-treated PKU is comparable to healthy controls [15–18]. In contrast, Huijbregts et al. (2018) showed that adults with early-treated PKU display alterations in HRQoL, particularly concerning cognition, anger, and depressive moods [19]. One of the reasons for these inconsistent findings can be explained by the different assessments to evaluate HRQoL [18]. Several studies use generic questionnaires to assess HRQoL which are likely not sensitive enough to capture subtle difficulties associated with PKU and dietary management [12, 18, 20]. For this reason, a PKU-specific QoL questionnaire has been developed, addressing four modules: symptoms associated with PKU, the impact of PKU, the impact of the dietary protein restriction on the patients’ everyday life, and the administration of the amino acid supplementation [21]. Studies using the PKU-QoL questionnaire suggest that most domains indicate little or no impact of PKU and its treatment on HRQoL, reflecting the good overall health status of adults with PKU [21]. However, some difficulties remain and patients report issues concerning the emotional impact of PKU and feelings of guilt if dietary restrictions are not followed, the taste of the supplements, the anxiety of high Phe levels during pregnancy, or tiredness [1, 18, 21, 22].

Previous studies investigating HRQoL using the PKU-QoL often included small and mixed samples of patients with hyperphenylalaninemia, mild, moderate, and classical PKU, samples of early-treated and non-early treated patients (treated before vs. after 30 days of age), as well as patients treated with sapropterin or pegvaliase [18, 22–25]. Adults with mild PKU treated with sapropterin displayed a higher HRQoL than adults with classical PKU treated with a Phe-restricted diet [25]. More specifically, patients with classical PKU reported a greater impact of the amino acid supplementation compared to patients with mild or moderate PKU [18]. Also, the overall impact of dietary protein restriction and the impact of the amino acid supplementation was higher for patients not treated with sapropterin than those treated with sapropterin [18]. These results reflect the increased dietary constraints for adults with classical PKU and highlights the importance of focusing on adults with early-treated classical PKU treated with dietary restriction and amino acid supplementation [18].

To date, factors associated with HRQoL in adults with early-treated PKU have been insufficiently studied. Male patients, patients with lower education and patients with higher concurrent Phe levels displayed lower HRQoL [19, 23, 25, 26]. However, previous findings should be interpreted with caution due to the inclusion of heterogeneous samples including patients with classical and non-classical PKU, pediatric and adult patients with PKU, and the use of divergent QoL assessments [19, 23, 25, 26]. In healthy adults, HRQoL has been shown to vary in respect to age, with increasing age negatively affecting HRQoL [27]. Also, higher intelligence quotients (IQ) and executive functions are linked to higher HRQoL in healthy adults [28, 29]. Whether age, IQ, and executive functions are related to HRQoL in adults with early-treated classical PKU remains to be investigated. Identifying patient characteristics associated with lower HRQoL is essential to provide the best medical care for this patient group.

The aim of this study was to investigate HRQoL in a large European sample of adults with early-treated classical PKU not treated with sapropterin or pegvaliase. We further aimed to explore associations between HRQoL and demographic, metabolic, and cognitive characteristics to extend our understanding of HRQoL in adults with early-treated classical PKU and to identify patients at risk for reduced HRQoL.

## Methods

### Study design and participants

This cross-sectional study includes data from five research projects that were carried out in Hungary, Italy, Spain, Switzerland [30], and Turkey. A subset of the data has previously been published [23, 24, 31]. In detail, data was prospectively collected between 2016 and 2022 at the Department of Internal Medicine and Oncology of the Semmelweis University in Budapest (Hungary), the Division of Inherited Metabolic Diseases of the University Hospital in Padova (Italy), the Unit of Diagnosis and Treatment of Congenital Metabolic Diseases of the University Clinical Hospital in Santiago de Compostela (Spain), the Department of Diabetes, Endocrinology, Nutritional Medicine and Metabolism of the University Hospital in Bern (Switzerland), and the Department of Nutrition and Dietetics of the Ankara University in Ankara (Turkey). Patients were recruited via their treating specialist or patient organizations. Details about the recruitment process have been previously described [23, 24, 30, 31]. The studies were approved by the local ethic committees of Hungary, Italy, Spain, Switzerland, and Turkey and were performed in accordance with the Declaration of Helsinki. All participants gave written informed consent before participation.

Participants included in this study were aged ≥ 18 years, diagnosed with early-treated classical PKU after a positive newborn-screening, and treated with a Phe-restricted diet and amino acid supplements according to the current guidelines [32]. Exclusion criteria were a treatment with sapropterin dihydrochloride (BH4, Kuvan®) or pegvaliase (Palynziq®). 173 participants were identified from the five research projects. In total, 49 patients were ineligible and therefore not included in the present study. 28 patients did not have classical PKU, 12 patients were not early-treated, and nine patients were treated with either sapropterin or pegvaliase. The final sample consisted of 124 adults with early-treated classical PKU.

### Outcomes

#### Health-related quality of life

The PKU-QoL is a disease-specific questionnaire designed to evaluate the impact of PKU and its treatment on HRQoL [21]. In contrast to more generic assessments of HRQoL, such as the EQ-5D [33], SF-12 [34], or SF-36 [35], the PKU-QoL is sensitive in terms of detecting specific difficulties related to PKU [18]. All participants completed the adult version of the PKU-QoL. This questionnaire consists of 65 items and allows the calculation of 35 domain scores across four modules (symptoms, PKU in general, supplement administration, and dietary protein restriction). Domain scores range from 1 to 100 whereby scores ≤ 25 reflect little or no impact, domain scores between 26 and 50 suggest moderate impact, domain scores between 51 and 75 indicate major impact, and domain scores > 75 reflect severe impact [18]. The PKU-QoL was scored using the PKU-QoL electronic scorer (PKU-QOL © Biomarin Pharmaceutical Inc.).

We defined two variables of particular interest in this study: “overall impact of PKU” and “overall impact of dietary protein restriction”. In contrast to most of the single-item domain scores of the PKU-QoL, these are two multi-item domain scores and include 13 items each. The domain score “overall impact of PKU” reflects the emotional, practical, and social impact of PKU (item example: “PKU negatively impacts my relationship with my partner”). The domain score “overall impact of dietary protein restriction” reflects the practical and social implications of dietary protein restriction (item example: “In the past 7 days, I felt different because I couldn’t eat or drink what others ate”). Items are scored on a 5- or 6-point Likert scale [21]. As the impact of PKU on HRQoL can be determined by cultural factors, a cross-cultural adaptation was performed for seven countries (Germany, France, Italy, Spain, the Netherlands, Turkey, and UK) during the development of the PKU-QoL [21].

#### Demographic, metabolic, cognitive, and psychosocial data

Demographic data (age and sex) were collected at the study visit. Information about Phe levels was available for the Hungarian, Italian, Spanish, and Swiss sample. Concurrent Phe levels were obtained from either blood plasma or dried blood spots. We additionally calculated the mean Phe levels of the past year (except for the Spanish sample, where we had information about the past two years). Plasma Phe was measured using high-performance ion-exchange liquid chromatography, and Phe levels in dried blood spots were assessed with tandem mass spectrometry in the laboratories of the study centers in the respective country. A calibration factor was not applied to adjust for discrepancies between Phe levels obtained from blood plasma or dried blood spots due to large variability between analytical methods [36]. IQ was integrated as a broad cognitive measure describing the patients’ general cognitive state. Furthermore, we chose to focus on IQ as this was the only cognitive outcome available in several samples (Swiss and Spanish sample). In the Swiss sample, IQ was assessed using a short form of the Wechsler Adult Intelligence Scale (WAIS-IV; [37, 38]) at the time of the HRQoL assessment. In the Spanish sample, IQ was assessed using the Kaufmann Brief Intelligence Test (KBIT; [39]) or the Wechsler Intelligence Test for Children (WISC-IV; [40]) as part of the clinical routine and was performed before the HRQoL assessment (age at the time of IQ assessment 13.82 years ± 2.14).

For a subgroup (Swiss sample, *n* = 30), information about performance in executive function and depressive symptoms was available. Executive functions were assessed using the theoretical framework of Miyake et al. (2000) [41]. In detail, working memory was measured using the subtest letter-number sequencing of the WAIS-IV, inhibition was evaluated using the third condition of the Color-Word Interference Test (CWIT) of the Delis-Kaplan Executive Function Test (D-KEFS; [42]), and cognitive flexibility was assessed using the fourth condition of the CWIT. According to the manuals, raw scores were transformed into age-corrected scaled scores (mean: 10 ± 3). We calculated the mean score of the three executive function domains to obtain a composite score of executive functions [43]. The Beck-Depression Inventory (BDI-II; [44]) was administered to assess depressive symptoms. This self-administered questionnaire consists of 21 items evaluating the level of depression with a score ranging from 0 to 63.

### Statistical analysis

To reduce the number of variables for the statistical analyses, we chose two out of the 35 domain scores of the PKU-QoL that were of particular interest, namely the multi-item domains “overall impact of PKU” and “overall impact of dietary protein restriction”. We did not compare the five European samples with respect to demographic, metabolic, and cognitive characteristics or HRQoL, given that the sample size sample varied considerably. Non-parametric statistics were used as not all variables were normally distributed. Categorical variables are displayed in frequencies and percentages, and continuous variables in medians and interquartile ranges (IQR). Associations between demographic, metabolic, cognitive variables, and the PKU-QoL were examined with two-sided Spearman correlations or Mann-Whitney *U*-tests. To further investigate the relationship between demographic, metabolic, and cognitive variables, a multiple linear regression was conducted to examine the variance of the dependent variable (“overall impact of PKU” and “overall impact of dietary protein restriction”, respectively) that is explained by the independent variables (age, sex, concurrent Phe levels, and IQ). Concurrent Phe levels and not Phe levels of the past year were chosen as an independent variable because more data was available for concurrent Phe levels.

Effect sizes *r* were calculated for Spearman correlations and Mann-Whitney *U*-tests and Cohens *f*^2^ were computed for multiple regressions. Effect sizes are interpreted according to Cohen [45] with small effect for *r* = .1 and *f*^2^ = 0.02, medium effect for *r* = .3 and *f*^2^ = 0.15, and large effect for *r* = .5 and *f*^2^ = 0.35. Further, 95% confidence intervals (CI) are reported for effect sizes to estimate the size of the effect rather than emphasizing statistical significance [46, 47]. To account for multiple testing, the false discovery rate (FDR) correction was applied [48]. Statistical analysis was performed with SPSS, version 28. Data visualization was conducted using the R package ggplot2 [49].

## Results

Demographic, metabolic, and cognitive data are presented in Table 1. Concurrent Phe levels were available for 108 participants (87.1%), and Phe levels from the past year before study participation were available for 95 participants (76.6%). 71 participants (65.7%) had higher concurrent Phe concentrations than suggested by the current European guidelines, which recommend maintaining a Phe level below 600 μmol/l [32]. Regarding the Phe levels during the year before study participation, 54 participants (56.8%) had a mean Phe level higher than 600 μmol/l. IQ was available for 37 participants (29.8%) from Spain and Switzerland, and the median IQ was 97.0 and hence within the normative range.

**Table 1:** Demographic, metabolic, and cognitive data

	Total *n* = 124	Hungary *n* = 66	Italy *n* = 5	Spain *n* = 7	Switzerland *n* = 30	Turkey *n* = 16
**Age**, years	30.0 (22.5–37.6)	32.5 (24.6–40.1)	30.0 (22.0–34.5)	30.6 (20.7–37.4)	35.5 (25.0–38.2)	22.0 (20.3–23.0)
**Sex**, male	59 (47.6%)	35 (53.0%)	2 (40.0%)	2 (28.6%)	17 (56.7%)	3 (18.8%)
**Concurrent Phe**, μmol/l	703.7 (540.0–907.8)	643.0 (503.8–835.0)	1073.0 (738.4–1411.0)	705.2 (614.4–1107.8)	741.0 (583.5–959.0)	-
**Phe past year**, μmol/l	650.0 (507.7–854.8)	598.3 (485.6–798.2)	1004.0 (677.7–1252.0)	696.2 (641.7–783.9)	813.3 (576.6–1053.7)	-
**IQ**	97.0 (90.0–106.0)	-	-	95.0 (81.0 -101.0)	97.0 (90.0–107.5)	-

*Notes*. Data are presented in frequencies (%) for categorical variables and in median (IQR) for continuous variables. Concurrent Phe available for *n* = 108, Phe past year for *n* = 95, and IQ for *n* = 37

### Health-related quality of life

Table 2 shows the 35 domain scores of the PKU-QoL. 27 of the 35 domain scores (77.1%) showed no or little impact of PKU (scores between 0 and 25). The four most affected domains were “tiredness”, “anxiety - Phe levels during pregnancy”, “taste - supplements”, and “guilt if dietary protein restriction not followed”. Except for the domain “anxiety - Phe levels during pregnancy,“ which was estimated to be a major impact, the other three dimensions were reported as moderate. For the domain “self-rated health status”, a median score of 50 suggests a moderate impact. This is misleading insofar as a score of 50 in this domain indicates that patients rated their overall health status as good. We therefore prefer to report the following: 1.7% of patients rated their overall health status as poor, 20.7% as fair, 28.9% as good, 28.1% as very good, and 20.7% as excellent.

**Table 2:** Health-related quality of life

Module	Domain	Total *n* = 124	Hungary *n* = 66	Italy *n* = 5	Spain *n* = 7	Switzerland *n* = 30	Turkey *n* = 16
Symptoms	Self-rated health status	50 (25–50)^3^	50 (25–75)^3^	50 (25–63)	50 (25–75)	25 (0–50)	50 (25–50)
	Headaches	25 (0–50)^2^	25 (0–50)^2^	25 (13–50)	25 (0–50)	25 (0–50)	25 (0–50)
	Stomach Aches	0 (0–25)^3^	0 (0–25)^3^	25 (0–50)	0 (0–50)	0 (0–25)	25 (0–25)
	*Tiredness*	*50 (25–50)* ^*2*^	*50 (25–50)* ^*2*^	*50 (13–75)*	*50 (50–75)*	*25 (0–50)*	*38 (7–69)*
	Lack of concentration	25 (0–50)^3^	25 (0–50)^3^	50 (13–75)	50 (25–50)	25 (0–50)	25 (0–44)
	Slow thinking	0 (0–25)^2^	0 (0–44) ^2^	25 (0–63)	25 (0–50)	0 (0–25)	13 (0–25)
	Trembling hands	0 (0–25)	0 (0–25)	0 (0–50)	0 (0–25)	0 (0–0)	0 (0–25)
	Irritability	25 (0–50)^2^	25 (0–50)^2^	25 (13–63)	25 (25–50)	25 (0–50)	25 (6–25)
	Aggressiveness	0 (0–25)^1^	0 (0–25)^1^	0 (0–50)	25 (0–50)	0 (0–0)	0 (0–19)
	Moodiness	25 (0–50)	25 (0–50)	25 (25–75)	50 (50–100)	13 (0–31)	38 (25–50)
	Sadness	0 (0–50)^1^	0 (0–50)^1^	25 (13–50)	25 (25–50)	0 (0–6)	38 (25–50)
	Anxiety	0 (0–50)^1^	25 (0–50)^1^	25 (0–63)	50 (50–75)	0 (0–0)	25 (0–50)
PKU in general	Emotional impact of PKU	35 (20–50)^3^	35 (20–50)^3^	40 (35–63)	50 (45–60)	20 (15–30)	40 (35–60)
	Practical impact of PKU	13 (0–25)^18^	13 (0–25)^5^	19 (4–29)	31 (25–31)	8 (6–18)	- ^13^
	Social impact of PKU	11 (0–19)^2^	7 (0–19)^2^	19 (15–25)	25 (19–31)	6 (0–13)	17 (8–25)
	**Overall impact of PKU**	**21 (12–31)** ^**4**^	**21 (13–30)** ^**4**^	**25 (23–42)**	**37 (33–42)**	**13 (10–17)**	**35 (20–44)**
	Anxiety - Blood test	0 (0–0)^7^	0 (0–13)^7^	0 (0–13)	0 (0–50)	0 (0–0)	0 (0–10)
	Anxiety - Phe levels	25 (25–75)^1^	25 (0–63)^1^	50 (50–63)	75 (75–100)	25 (25–31)	50 (25–75)
	*Anxiety - Phe levels during pregnancy*	*75 (25–100)*	*50 (25–100)*	*75 (50 - NA)*	*100 (75–100)*	*75 (25–88)*	*75 (25–100)*
	Financial impact of PKU	25 (0–50)^1^	0 (0–25)^1^	0 (0–13)	75 (50–100)	25 (0–31)	25 (0–50)
	Information on PKU	25 (0–50)^3^	25 (0–50)^3^	50 (13–50)	50 (25–50)	25 (0–25)	25 (0–44)
Supplement administration	Adherence to supplements	8 (2–33)^28^	8 (0–31)^14^	50 (33–63)	8 (0–25)	8 (4–25)^1^	- ^13^
	Guilt if poor adherence to supplements	25 (25–75)^6^	38 (25–75)^6^	50 (25–50)	25 (0–25)	25 (25–50)	25 (25–50)
	Impact of supplements on family	0 (0–0)^5^	0 (0–0)^5^	0 (0–50)	0 (0–0)	0 (0–0)	0 (0–25)
	Practical impact of supplements	6 (0–25)^7^	0 (0–13)^7^	25 (13–31)	38 (31–44)	6 (0–13)	38 (19–69)
	*Taste - Supplements*	*50 (25–50)* ^*8*^	*50 (25–50)* ^*8*^	*50 (50–63)*	*50 (25–50)*	*25 (25–50)*	*50 (6–50)*
Dietary protein restriction	Food temptation	32 (0–50)^6^	25 (0–50)^5^	63 (44–75)	50 (25–50)	25 (13–50)^1^	19 (0–50)
	Adherence to dietary protein restriction	19 (6–30)^17^	19 (10–34)^10^	35 (28–58)	25 (13–25)	13 (5–25)^1^	25 (6–33)^6^
	Practical impact of dietary protein restriction	29 (15–39)^9^	25 (11–36)^8^	39 (27–54)	39 (25–50)	21 (11–34)^1^	54 (39–75)
	Social impact of dietary protein restriction	4 (0–13)^6^	5 (0–13)^6^	25 (15–38)	4 (0–33)	0 (0–4)	13 (5–29)
	**Overall impact of dietary protein restriction**	**17 (10**–**29**)^**9**^	**17 (9–25)** ^**8**^	**29 (23–46)**	**27 (13–52)**	**13 (7–18)** ^**1**^	**35 (29–46)**
	Overall difficulty following dietary protein restriction	25 (0–50)^7^	25 (0–50)^7^	50 (25–75)	0 (0–50)	25 (0–25)	50 (0–50)
	*Guilt if dietary protein restriction not followed*	*50 (25–75)* ^*6*^	*50 (25–75)* ^*6*^	*50 (25–63)*	*75 (50–75)*	*25 (25–56)*	*75 (38–94)*
	Taste - Low-protein food	25 (25–50)^9^	25 (25–50)^6^	50 (50–50)^1^	25 (25–25)	25 (0–25)^2^	25 (0–50)
	Food enjoyment	0 (0–25)^8^	0 (0–25)^6^	75 (50–75)	0 (0–25)	0 (0–0)	25 (25–88)

*Notes*. All values were rounded to the nearest integer. Number of missing data is indicated in superscripted numbers. Four most affected domains are presented in italic. The two domains of interest in this study are presented in bold. Domain scores are interpreted as follows: ≤ 25 = little or no impact; 26–50 = moderate impact; 51–75 = major impact; > 75 = severe impact (18). NA = Not available due to the limited number of female participants, *n* = 3

**Fig. 1:**
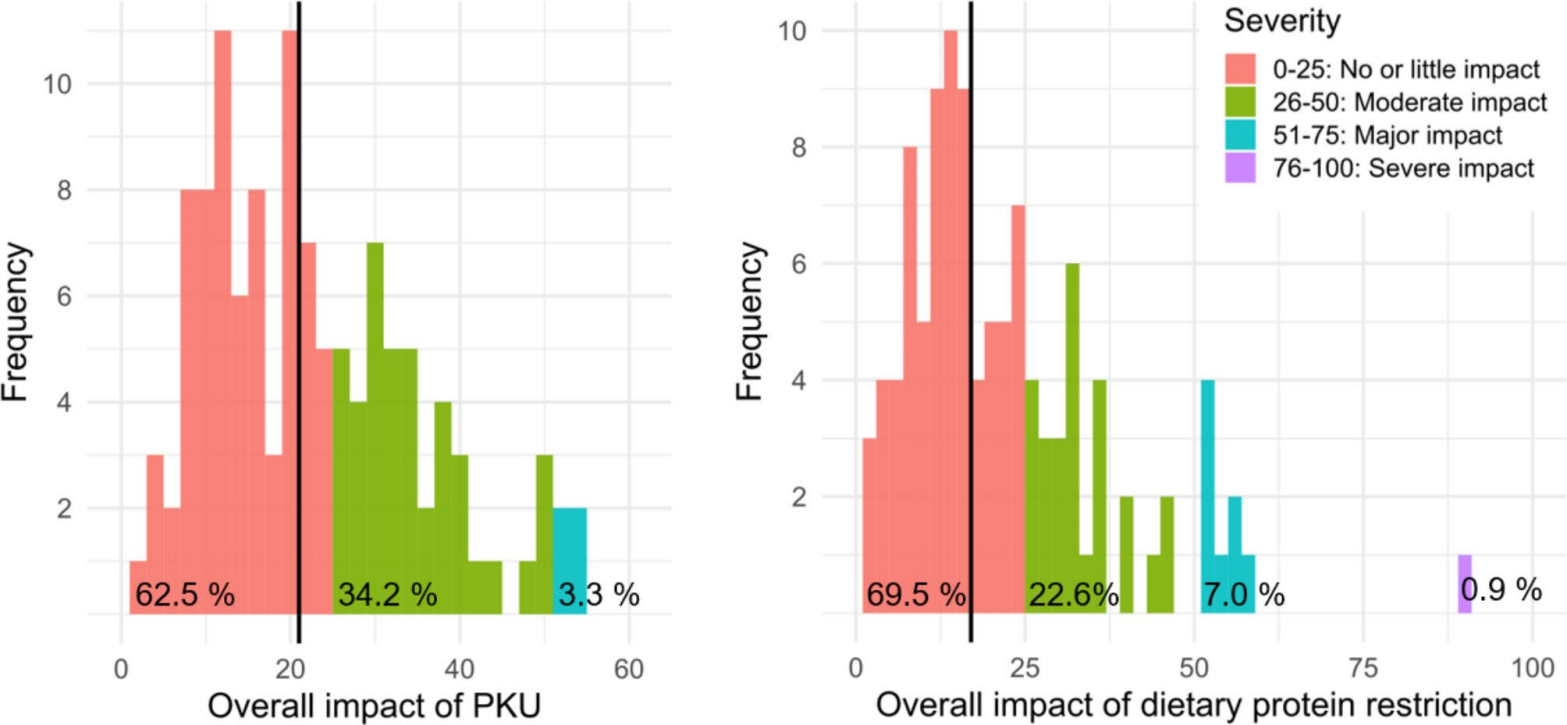
Distribution of the two domain scores overall impact of PKU and overall impact of dietary protein restriction. The vertical black line is the median. The percentages reflect the proportion of participants in the corresponding category

The median scores of “overall impact of PKU” and “overall impact of dietary protein restriction” were 21 and 17, respectively, which suggest no or little impact (bold domains in Table 2). Detailed information about the distribution of the data is displayed in Fig. 1. Although the median scores were both interpreted as no or little impact, a considerable part of the patients reported a moderate or major impact of PKU or dietary protein restriction, respectively. These two domain scores were considered for further statistical analyses.

### Associations between health-related quality of life and demographic, metabolic, and cognitive characteristics

**Fig. 2:**
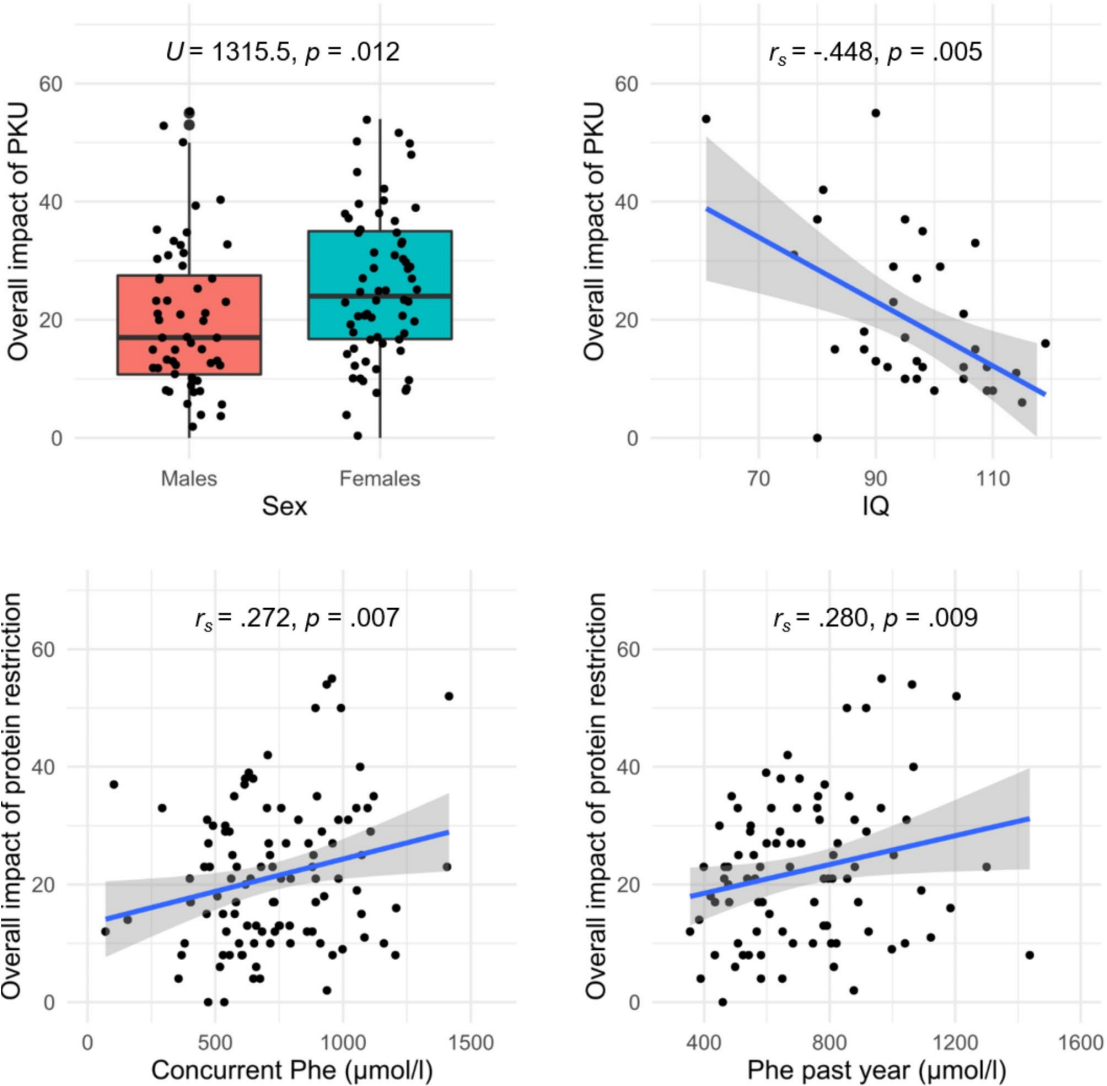
Significant associations between overall impact of PKU / overall impact of dietary protein restriction and demographic, metabolic, and cognitive characteristics (surviving FDR correction). The blue line represents the correlation coefficient with 95% CI in grey. A boxplot is presented for the categorical variable sex. *p* = significance value, *r*_*s*_ = Spearman’s correlation coefficient, *U* = Mann-Whitney *U*

The overall impact of PKU was not significantly associated with age (*r*_*s*_ = − 0.125, *p* = .173, 95% CI [-0.30, 0.06]). Women (median = 24, IQR = 16–35) rated the overall impact of PKU significantly higher compared to men (median = 17, IQR = 10–29; *U* = 1315.5, *p* = .012, *r* = .23, 95% CI [0.05, 0.39]; see also Fig. 2, top left). This comparison survived FDR correction (*p*_*FDR*_ = 0.032). Concurrent Phe levels (*r*_*s*_ = 0.196, *p* = .046, 95% CI [0.00, 0.38]) but not Phe levels of the past year (*r*_*s*_ = 0.167, *p* = .114, 95% CI [-0.04, 0.36]), were significantly associated with the overall impact of PKU, which, however, did not persist after FDR correction (*p*_*FDR*_ = 0.092). A significant negative association with medium effect sizes was found for overall impact of PKU and IQ (*r*_*s*_ = − 0.448, *p* = .005, 95% CI [-0.68, -0.13]), with higher overall impact of PKU relating to lower IQ (Fig. 2, top right). This association survived FDR correction (*p*_*FDR*_ = 0.027).

A multiple linear regression with age, sex, concurrent Phe, and IQ as independent variables and overall impact of PKU as dependent variable showed that 18.6% of the variance in the overall impact of PKU could be explained by the independent variables (*F* [4,32] = 3.051, *p* = .031, *f*^2^ = 0.23, 95% CI [-0.01, 0.62]). IQ was a significant predictor (*p* = .005) whereas age (*p* = .891), sex (*p* = .531), and concurrent Phe (*p* = .306) were not. After FDR correction, the regression model was not significant (*p*_*FDR*_ = 0.071).

The overall impact of dietary protein restriction was neither significantly correlated with age (*r*_*s*_ = − 0.111, *p* = .237, 95% CI [-0.29, 0.07]) nor IQ (*r*_*s*_ = − 0.076, *p* = .658, 95% CI [-0.40, 0.26]). There was no sex difference (men median = 17, IQR = 10–26; women median = 19, IQR = 11–33) regarding the overall impact of dietary protein restriction (*U* = 1509.0, *p* = .439, *r* = .07, 95% CI [-0.11, 0.25]). Concurrent Phe levels (*r*_*s*_ = 0.272, *p* = .007, 95% CI [0.08, 0.45]) and Phe levels of the past year (*r*_*s*_ = 0.280, *p* = .009, 95% CI [0.07, 0.47]) were significantly associated with the overall impact of dietary protein restriction, with higher Phe levels relating to higher overall impact of dietary protein restriction (Fig. 2, bottom row). Significant results survived FDR correction (*p*_*FDR*_ = 0.028 and *p*_*FDR*_ = 0.029) with small effect sizes. The multiple regression model with overall impact of dietary protein restriction as dependent variable was not significant (*F* [4,31] = 0.442, *p* = .777, *f*^2^ = 0.00).

### Subgroup analyses

**Fig. 3:**
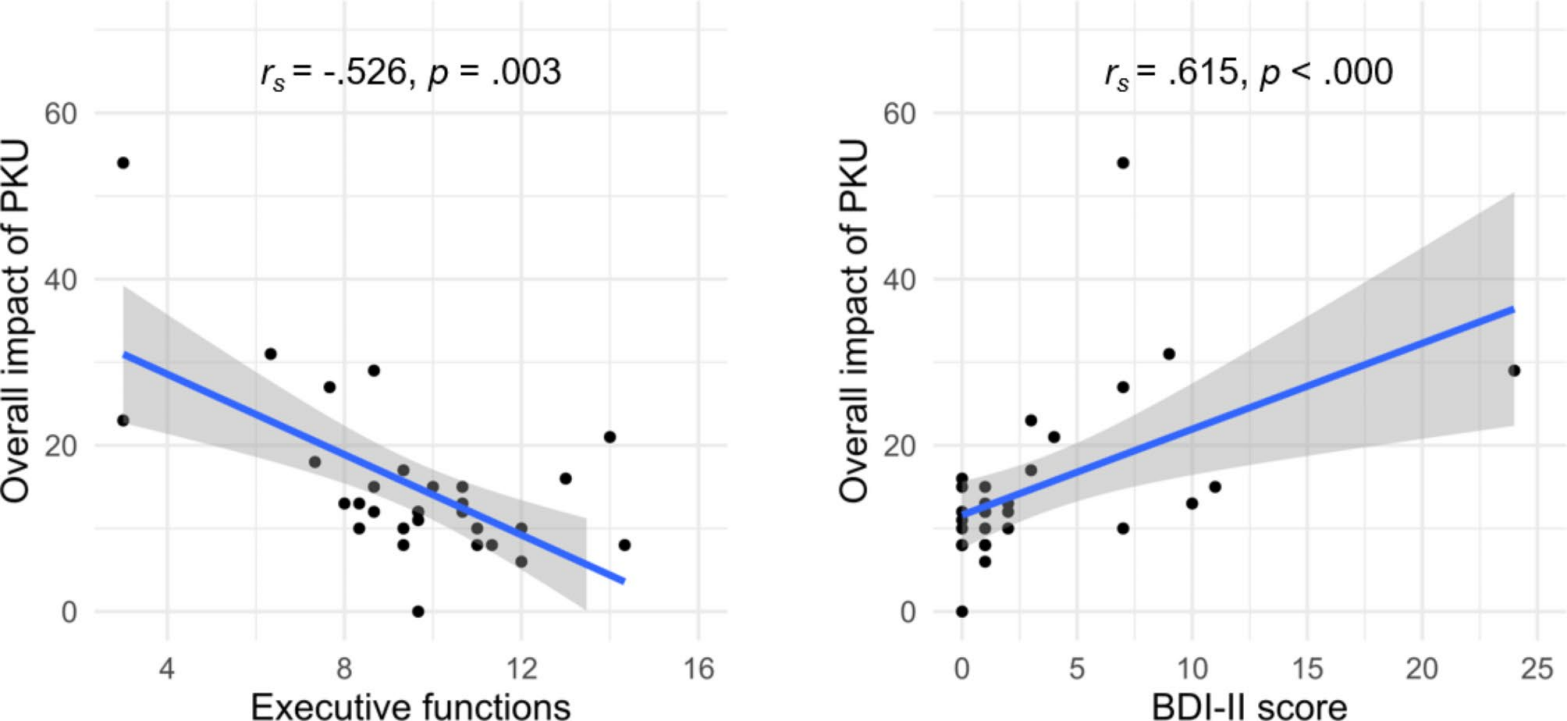
Associations between the overall impact of PKU and executive functions and the depression score in the Swiss subsample (n = 30). The blue line represents the correlation coefficient with 95% CI in grey. Results remained the same when the outlier (BDI-II score of 24) was excluded. *p* = significance value, *r*_*s*_ = Spearman’s correlation coefficient

Significant results of the subgroup analyses in the Swiss sample are displayed in Fig. 3. The median (IQR) composite score for executive functions was 9.7 (8.3–11). There was a significant negative association between the overall impact of PKU and executive functions (*r*_*s*_ = − 0.526, *p* = .003, 95% CI [-0.76, -0.18]) with a large effect size, indicating that patients with a high overall impact of PKU displayed worse executive functions. The median (IQR) depression score was 1 (0–5.5). A significant positive correlation with a large effect size was found between the overall impact of PKU and the depression score (*r*_*s*_ = 0.615, *p* < .000, 95% CI [0.30, 0.81]), with a high overall impact of PKU relating to a higher depression score. Findings of the subgroup analyses remained significant after FDR-correction (*p*_*FDR*_ = 0.024 and *p*_*FDR*_ < 0.000). The overall impact of dietary protein restriction was neither significantly related to executive functions (*r*_*s*_ = 0.079, *p* = .685, 95% CI [-0.30, 0.43]) nor to the depression score (*r*_*s*_ = 0.327, *p* = .083, 95% CI [-0.06, 0.63]).

## Discussion

In this cross-sectional European study including 124 adults with early-treated classical PKU, we showed that the majority of the domains assessed with the PKU-QoL indicated no or little impact of PKU on HRQoL. More than three-quarters of the patients rated their health status as good, very good, or excellent. However, participants were worried about high Phe levels during pregnancy and were concerned about the guilt if dietary protein restriction was not followed. Tiredness was the most affected symptom and the supplements’ taste was considered a main issue for individuals with PKU. The medians of overall impact of PKU and overall impact of dietary protein restriction were considered to be low. Nevertheless, the overall impact of PKU was higher in females and participants with a lower IQ. The overall impact of protein restriction was higher in patients with higher concurrent Phe levels and higher Phe levels during the past year.

77.1% of the domain scores assessed in the PKU-QoL questionnaire showed no or little impact of PKU on HRQoL and a majority of the participants rated their health status as good, very good, or excellent. This is consistent with previous studies [15, 16, 23] and likely reflects the successful implementation of the newborn screening resulting in the prevention of severe adverse long-term outcomes. Similar to other chronic or serious diseases, where patients often report a good HRQoL, adults with early-treated classical PKU likely undergo positive adaptation processes and thus show good HRQoL [12, 50]. Of note, it is important to consider that most participants were recruited via their treating specialist at their metabolic clinic or via patient organizations, which could bias our sample. Some adults with PKU are lost to follow-up by metabolic clinics and are probably not adhering to a life-long diet [51, 52]. This circumstance is also reflected in a large survey including adults with PKU recruited via websites and social media, which suggested that social isolation and stigmatization associated with dietary management represent a burden for patients [7]. Thus, the results of the present study likely refer to patients who are followed up by a metabolic clinic and may not be applicable to all patients.

Despite the overall good outcome in terms of HRQoL in adults with early-treated PKU, some areas of concern have been identified in the present study. Future research should particularly focus on the taste of the amino acid supplements, as this was a significant issue for patients, which can negatively impact compliance to the supplementation [53]. The taste of the ready-to-use amino acid supplements is continuously being improved, however, to date full reimbursement of these confectioned products by health insurance is not guaranteed in some countries [54]. Also, patients were concerned about the guilt that they experience if they do not adhere to the dietary protein restriction. Dietary management is often perceived as stressful and represents a burden for patients, which likely leads to difficulties associated with the adherence to the current dietary recommendations [55–57]. In fact, PKU-related symptoms but also dietary management itself can negatively affect HRQoL [12]. There is still no international consensus on ideal Phe concentrations during adulthood [56]. The current European guidelines suggest maintaining a Phe level below 600 μmol/l [32], but this has been criticized due to limited evidence for safe target Phe concentrations and the difficulties for patients to reach these treatment goals [56, 58]. Our data show that patients feel guilty if they do not follow their diet. This further points to the idea that overtreatment might negatively impact their psychological well-being with no or limited potential benefits [56]. It is therefore of crucial importance to investigate in a randomized controlled trial whether high Phe levels affect HRQoL, cognitive, and cerebral parameters in adults with early-treated PKU to increase our understanding of safe target Phe levels for adults with early-treated classical PKU [30].

Investigating patient characteristics associated with HRQoL is essential to identify patients at risk for lower HRQoL and consequently provide the best medical care. Regarding the overall impact of PKU, our data show that women displayed a higher overall impact of PKU than men. This is in contrast to a study with 17 adults with PKU, suggesting worse HRQoL in men compared to women [25]. These conflicting results can be explained by the small and heterogeneous sample in the previous study, as they included patients with mild and classical PKU and patients treated with sapropterin. The sex difference regarding the overall impact of PKU found in the present study is, however, in line with studies in other clinical populations suggesting that women generally report worse HRQoL than men [59–63]. Women differ from men in their health perception and behavior and how they report symptoms [64–66], all of which can influence the self-reported HRQoL. Also, the fear of maternal PKU and its teratogenic effects during prenatal development could also contribute to a higher impact of PKU on HRQoL in women compared to men [67]. Further, differences in socioeconomic status (SES) might contribute to the sex difference observed in the present study [68]. Women tend to have lower incomes than men; thus, sex disparities in HRQoL can also partly be attributed to the lower SES of women [68]. We were unable to include SES in the present study and examine its association with HRQoL because of difficulties in comparing income and educational levels across countries. To overcome this, future studies should implement a standardized assessment to evaluate SES, such as the Hollingshead index [69].

Our findings further suggest that the overall impact of PKU is lower in participants with a higher IQ. Given the non-directional relationship of this association, it can be argued that a higher IQ is a protective factor that might protect against the negative impact of PKU [70]. At the same time, PKU adversely affects IQ, and lower IQ has been observed in adults with poorer metabolic control [71, 72]. Further, we found a positive association between the overall impact of dietary protein restriction and concurrent Phe levels as well as Phe levels of the past year. Maintaining a strict diet in classical PKU can be a demanding and sometimes frustrating task. Particularly in adulthood, with growing personal independence and after years of strict diet, these results could reflect the patients’ resentment towards a strict diet and maintaining low Phe levels. Although the impact of PKU on HRQoL was considered to be low in the majority of the domains assessed in this study, specific patient characteristics are associated with lower HRQoL in adults with early-treated classical PKU. A particular focus should be given to patients with lower IQ, higher Phe levels, and women, as they are at risk for lower HRQoL.

We further examined in the Swiss subsample whether executive functions and depressive symptoms were related to the overall impact of PKU and the overall impact of dietary protein restriction. Executive functions and depressive symptoms were strongly associated with the overall impact of PKU but unrelated to the overall impact of dietary protein restriction. These results suggest that the overall impact of PKU is higher in patients with worse executive functions and higher depression scores. This is in line with studies including other clinical populations, suggesting that executive functions and depressive symptoms are associated with HRQoL [73–77]. The findings of the present study highlight the importance of focusing on cognitive and psychosocial factors in the treatment of PKU, enabling the identification of patients that are particularly vulnerable to impaired HRQoL.

A strength of the present study is the size and homogeneity of the European sample including 124 adults with early-treated classical PKU, a group of patients neither treated with sapropterin nor with pegvaliase. Further, our results show medium to large effect sizes that withstand correction for multiple testing. The study also has some limitations. First, we did not statistically compare the different national subsamples regarding their HRQoL as the sample sizes varied considerably across countries. Also, the included countries differ in their cultural backgrounds and health care systems – both aspects can influence HRQoL. However, the PKU-QoL questionnaire has been cross-culturally adapted for Germany, Italy, Spain, and Turkey to minimize the impact of these influences on HRQoL [21]. Of note, no cross-cultural adaptation of the questionnaire has been performed for Hungary (for more details see [23]). Second, IQ was assessed with two different measures. However, the literature suggests that IQ scores that are assessed with different measures are highly correlated [78], hence we start from the premise that comparability between IQ measures is given. Furthermore, IQ was only available in a subsample of 37 participants. In addition, in the Spanish sample (*n* = 7) IQ was assessed during childhood and thus, years before the HRQoL assessment. Previous studies on patients with PKU have shown stable cognitive functions across childhood and adulthood [79]. To find out whether the different age at IQ assessment influenced the association between IQ and HRQoL, analyses were re-performed with only the patients from the Swiss subgroup (*n* = 30). The results remained the same, showing a significant association between IQ and overall impact of PKU. To conclude, the association between IQ and HRQoL should be interpreted in light of this limitation. Further, IQ is a general measure not sensitive enough to detect minor cognitive alterations, especially in adult patients. Third, comparability of the disease-specific HRQoL questionnaire to other HRQoL instruments or to HRQoL in the healthy population is limited as the PKU-QoL questionnaire is a PKU-specific instrument. At the same time, the specificity of the PKU-QoL questionnaire is a strength of this study in identifying areas of concern for patients with a particular focus on dietary aspects. The PKU-QoL questionnaire could ideally be complemented with the newly developed PKU Symptom Severity and Impacts Scale (PKU-SSIS) addressing neuropsychological and physical symptoms of PKU and its impact on HRQoL [80]. Fourth, additional variables that might be associated with HRQoL, for instance, the SES, were not included in the present study because of difficulties in comparing income and educational levels across countries.

## Conclusion

The present study shows that for more than three-quarters of the domains assessed in the PKU-QoL, the impact of early-treated classical PKU on HRQoL was considered to be low. These results reflect the successful implementation of the newborn screening preventing severe adverse long-term outcomes. However, our results reveal that a particular focus should be given to adults with a lower IQ, higher Phe levels, and women, as these characteristics were associated with a higher overall impact of PKU and dietary protein restriction, respectively. These findings further contribute to a better understanding of the complex nature of demographic, metabolic, and cognitive factors associated with HRQoL and show the importance of including patient-reported outcome measures to identify areas of particular concern for patients with classical PKU.

## Data Availability

The datasets used and analyzed during the current study are not publicly available in order to protect patient privacy. However, the datasets are available from the corresponding author (regula.everts@insel.ch) upon reasonable request.

## List of abbreviations

BDI-II: Beck Depression Inventory
BH4: Sapropterin dihydrochloride
CWIT: Color-Word Interference Test
CI: Confidence interval
D-KEFS: Delis-Kaplan Executive Function Test
EQ-5D: European Quality of Life 5 Dimensions
FDR: False discovery rate
HRQoL: Health-related quality of life
IQ: Intelligence quotient
IQR: Interquartile range
K-BIT: Kaufmann Brief Intelligence Test
PAH: Phenylalanine hydroxylase
Phe: Phenylalanine
PKU: Phenylketonuria
PKU-QoL: Phenylketonuria quality of life questionnaire
SES: Socioeconomic status
SF-12: Short Form 12
SF-36: Short Form 36
WAIS-IV: Wechsler Adult Intelligence Scale
WISC-IV: Wechsler Intelligence Test for Children
